# Cell chirality exhibition of brain microvascular endothelial cells is dependent on micropattern width†

**DOI:** 10.1039/d2ra05434e

**Published:** 2022-10-21

**Authors:** Ana María Porras Hernández, Maria Tenje, Maria Antfolk

**Affiliations:** Dept. of Materials Science and Engineering, Science for Life Laboratory, Uppsala University Uppsala Sweden maria.tenje@angstrom.uu.se; Dept. of Biomedical Engineering, Lund University Lund Sweden maria.antfolk@bme.lth.se; Biotech Research and Innovation Centre, University of Copenhagen Copenhagen Denmark

## Abstract

Left-right asymmetry is a conserved property in nature and observed in the human body, a property known as cell chirality. Cell chirality is often studied using micropatterned *in vitro* models. However, micropattern geometry and size often varies across different studies, making it challenging to compare results. Here, we utilized micropatterned RGD-peptide lines on hyaluronic acid hydrogels to investigate the effect of the micropattern width on the exhibited cell chirality bias of brain microvascular endothelial cells. Overall, this cell type exhibited a negative chirality bias on micropatterned lines ranging from 10 μm to 400 μm in width, where the negative bias was most pronounced on the 100 μm wide lines. We also observed that this exhibited chirality bias varied across the line width. This work serves as a guide to determine optimal micropattern width for further investigations on cell chirality bias and its prominence in *e.g.*, disease states or upon exposure to toxic substances.

## Abstract 2

Brain endothelial microvascular cell chirality is dependent on micropattern width and spatial localization. Finding the optimal micropattern width and including only the centre cells in the analysis for chirality-based experiments might improve further experimental results.

## Graphical abstract

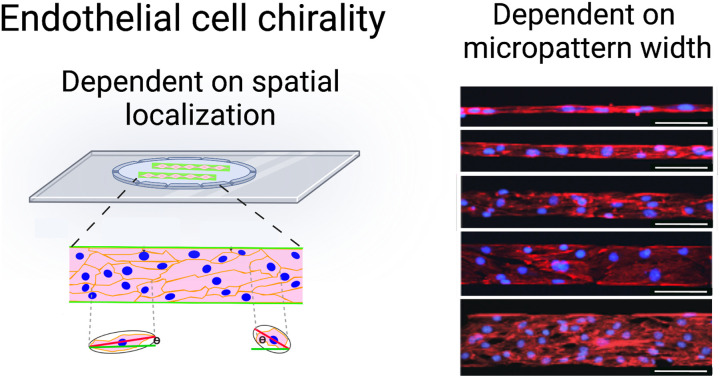

## Introduction

Externally, humans exhibit a bilateral symmetric body. However, on the inside, it is far from symmetric, where the organs’ shape and position present left-right asymmetry, *i.e.*, so-called chirality.^1,2^ Proper establishment of left-right asymmetry is required for normal embryonic development and function maintenance, and defects in asymmetry often result in birth defects.^3,4^ It has been proposed that cells possess an inherent chirality which translates into the formation of asymmetric tissue and organs.^2^

Cell chirality has been observed *in vivo*, but only in invertebrates.^5^ However, *in vitro* both vertebrates and human cell chirality has been observed, demonstrating an inherent cell chirality under specific culture conditions.^6–8^ A pioneering study by Wan *et al.* determined that cell chirality is cell type specific, when they were the first to observe that different cell types or disease states express different left-right orientation biases.^9^ This study has since been extended and this chirality bias has now been reported on various cell types (Table 1).

**Table 1:** Cell chirality varies depending on cell type and pathological state

Cell type	Bias	Species	Tissue	Phenotype	Cell origin	Pattern type	Pattern width (μm)	Ref
Human umbilical vein endothelial cells (HUVEC)	Negative	Human	Umbilical vein	Endothelial cells	Primary	Ring	200, 150, 125	^9^, ^13^ and ^23^, respectively
Human brain microvascular endothelial cells	Negative	Human	Brain capillary	Endothelial cells	Primary	Ring	150	^18^
Human skeletal muscle cells (hSkMC)	Positive	Human	Skeletal muscle	Myoblast	Cell line	Ring	200	^9^
C2C12	Positive	Mouse	Skeletal muscle	Myoblast	Cell line	Ring	200	^9^
C2C12	Negative	Mouse	Skeletal muscle	Myoblast	Cell line	Line	200	^15^ and ^16^
Rat cardiac fibroblast	Negative	Rat	Heart	Cardiac fibroblast	Primary	Ring	200	^9^
Human adipose stem cells (hASC)	Negative	Human	Adipose	Stem cells	Primary	Ring	200	^9^
Human mesenchymal cells (hMSC)	Negative	Human	Bone marrow	Stem cells	Primary	Ring	200	^9^
Human primary skin fibroblast	Negative	Human	Skin	Fibroblast	Primary	Ring	200	^9^
Human skin fibroblast line	Negative	Human	Skin	Fibroblast	Cell line	Ring	200	^9^
Human skin cancer fibroblast line	Positive	Human	Skin	Fibroblast	Cell line	Ring	200	^9^
NIH/3T3	Negative	Mouse	Embryo	Fibroblast	Cell line	Ring	200	^9^
MDCK	Positive	Dog	Kidney	Epithelial	Cell line	Ring	235	^19^
MDCK	Positive	Dog	Kidney	Epithelial	Cell line	Line	200	^14^
MC3T3-E1	Negative	Mouse	Calvaria	Osteoblast	Cell line	Ring	200	^9^

Micropatterned substrates have been used to study cell chirality *in vitro*, where cells have been cultured on well-defined boundaries in the micrometre scale to elucidate the mechanisms of cell chirality development.^7–10^ Micropatterned substrates are a suitable technology for such studies, as they provide a simple mean to achieve well-defined edges that are the required cue for the cells to display their chirality bias.^9,11^ Furthermore, the method can be used to produce a large number of microscale patterns to enable high-throughput studies with high statistical power.

Cell chirality offers an attractive comparative property of cells across cell studies due to its binary-switch nature.^9,12^ However, comparison between different studies is often complicated by the use of different micropattern geometries (*e.g.*, lines or rings) and different pattern widths.^9,13–18^ Typically, pattern widths between 100 and 300 μm have previously been used. Previous studies have reported that equivalent chirality bias was observed on both geometries, however no quantitative data was provided. Likewise, it has also been observed that there is no cell chirality bias exhibited on circle or square patterned geometries.^9^

From an analytical point of view, the determination of cell chirality based on alignment bias is more accessible on micropatterned lines, compared to rings. The analysis of cell orientation on ring micropatterns requires custom-made image processing algorithms in MATLAB or Python,^9,19^ as the *x*-axis changes depending on the ring location. On the other hand, cell orientation on micropatterned lines is straightforward as the *x*-axis is constant, and it can be performed in readily available image analysis software, such as CellProfiler™.^20^

Endothelial cells readily orient on micropatterned surfaces as well as *in vivo* where this exhibit promotes an atheroprotective phenotype.^21,22^ These cells have also been seen to exhibit a chirality bias. Previous studies have reported that endothelial cells exhibit a negative chirality bias on ring micropatterns. This was observed in human brain microvascular endothelial cells and in human umbilical vein endothelial cells (HUVECs) on ring patterns 150 μm or 200 μm wide, respectively.^9,18^ However, it is still largely unclear under which boundary conditions cells exhibit their chirality bias and at which pattern width this feature is most pronounced. Especially, micropatterns of widths less than 100 μm have been completely overlooked, even though blood vessels range from a couple of micrometers to centimeters in diameter, making their circumference in the range of 10 μm at their smallest.

In this paper, we used mouse brain microvascular endothelial cells to understand if these cells exhibit a cell chirality bias on micropatterned lines and investigate the effect of the different line widths on the cell chirality bias expressed. To find the optimal micropattern width where the cells exhibited the most pronounced chirality bias, we used RGD-peptide micropatterned hyaluronic acid hydrogels with lines of various widths (10–400 μm). Furthermore, we investigated the local expression of cell chirality across the width of the lines. When studying the effects of *e.g.*, disease states or toxic substances on cell chirality, where small changes, such as randomization or a reduced exhibited cell chirality bias might be the result, these small changes may go undetected on suboptimal micropattern sizes. We envision that our results can serve as a guide for further investigations on cell chirality.

## Results and discussion

### RGD-patterned hyaluronic acid hydrogels provide platform for cell chirality measurements

**Fig. 1:**
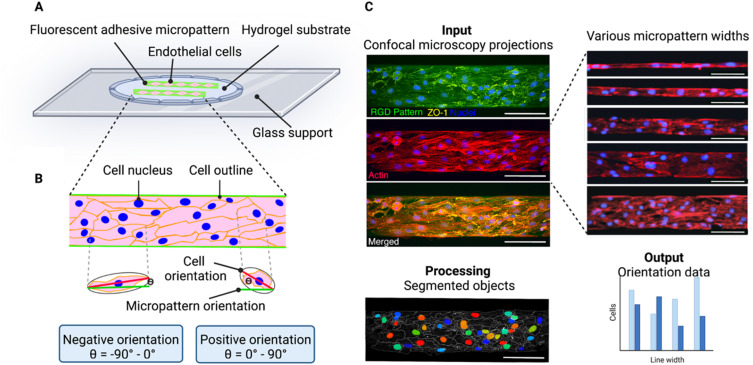
Experimental principle for determining cell chirality bias of brain endothelial cells on micropatterned substrate. (A) Schematic illustration showing endothelial cells adhered to fluorescent RGD-peptide micropatterns on hyaluronic acid hydrogel substrates. (B) Schematic illustration of cells on a line pattern illustrating the process of determining the cell orientation with respect to the line. An ellipse is fitted onto segmented cell from the ZO-1 image. The angle between the major axis (red) of the ellipse and the direction of the underlying RGD pattern (green) is calculated, ranging between ±90°. Cells with orientation values between −90° and 0° are positioned to the right and denoted negative orientation bias, and cells with an orientation between 0° and 90° are positioned to the left and have a positive orientation bias. The orientation of the nucleus or actin fibers are determined in a similar way. (C) The image analysis workflow. As input confocal maximum intensity projections of endothelial cells seeded on the micropatterned lines (here 100 μm wide) are used. The nuclei are shown in blue, ZO-1 depicting the cell outline in yellow, F-actin is shown in red, and the RGD pattern in green. To process the images CellProfiler™ is used to segment the cells (outlined in white) and identify the nuclei (in various colours). The output data for the chirality determination is the ratio of negatively *versus* positively oriented cells. Scale bars = 100 μm. Created with https://BioRender.com.

In this paper, we analyse cell chirality of brain microvascular endothelial cells on micropatterned lines. To do this, we seeded brain microvascular endothelial cells on various line micropatterns (Fig. 1A). Importantly, when cells are seeded too sparsely, random walk is likely to dominate over alignment bias, as the cells have more space to move.^14,24^ Hence, care was taken to find a seeding density where the cells completely covered the pattern.

Cell chirality can be defined as the biased cell orientation with respect to the micropattern direction, where we define the cells oriented between *Θ* = −90° and 0° as exhibiting a negative chirality bias and the cells orientated between *Θ* = 0° and 90° as exhibiting a positive chirality bias (Fig. 1B). To visualize the line patterns, we used a fluorescently conjugate RGD peptide. The cells were visualized staining the nucleus, zonula occludens 1 (ZO-1), and F-actin. To investigate the cell orientation with respect to the micropattern we used CellProfiler™, an open source image analysis software.^20^ Here, we inputted confocal maximum intensity projections and calculated the orientation of the cell body, nucleus, and actin fibres compared to the micropattern (Fig. 1C).

CellProfiler™ calculates the orientation of the cell by fitting the segmented cell to an ellipse. The orientation then corresponds to the angle between the *x*-axis (determined by the RGD pattern direction) and the major axis of the ellipse, giving values between ±90° (ref. 20) (Fig. 1B). A cell with its major axis positioned to the right will have a negative orientation (−90° to 0°) and a cell with its major axis positioned to the left, will have a positive orientation (0° to 90°) (Fig. 1B).

### Brain microvascular endothelial cells exhibit a negative chirality bias which is line width dependent

First, we wanted to determine the chirality bias of brain microvascular endothelial cells as well as investigate the boundary conditions for this chirality exhibition. Utilizing our hyaluronic acid hydrogels, we patterned these with RGD cell adhesion peptides in the form of lines with different widths ranging from 10 μm to 400 μm. As a control a non-patterned (NP) hydrogel was used where the whole hydrogel surface was functionalised with RGD-peptides.

**Fig. 2:**
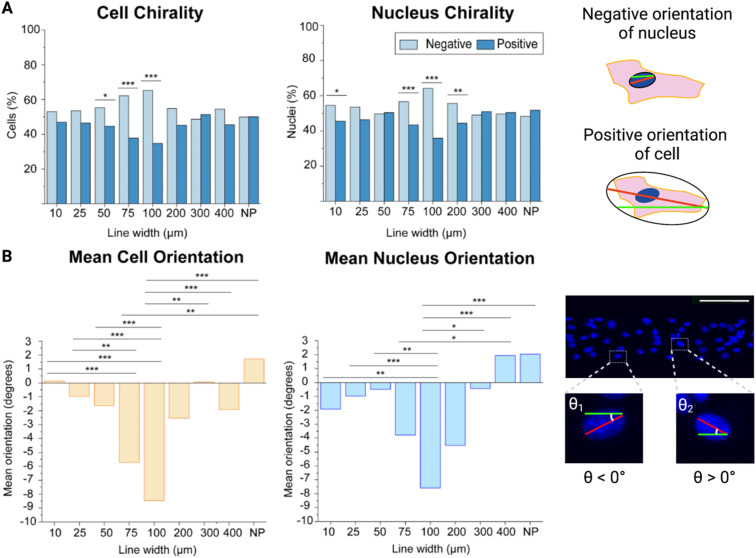
Cell and nucleus chirality of mouse brain microvascular endothelial cells on different line widths. (A) Left: bar plots with the percentage of cells and nuclei exhibiting a chirality bias (negative chirality bias in light blue and positive chirality bias in dark blue). Statistical analysis of cell and nucleus orientation of brain microvascular cells on different line widths compared to cells on a non-patterned surface was assessed using a chi-square test. Significance is symbolized by non-significant or N.S. (*p* > 0.05), * (*p* ≤ 0.05), ** (*p* value ≤ 0.01), *** (*p* value ≤ 0.001). Right: schematic illustration depicting a positively oriented cell and a negatively oriented nucleus compared to the green line. (B) Left: bar plots of the mean orientation of the cells and nuclei on the different line widths. Statistical analysis of cell and nucleus chirality of brain microvascular endothelial cells on different line widths and cells seeded on non-patterned hydrogels was determined using a one way analysis of variance (ANOVA) with a Games-Howell *post hoc* for multiple comparisons at a 95% confidence level. Significance is symbolized by non-significant or N.S. (*p* > 0.05), * (*p* ≤ 0.05), ** (*p* value ≤ 0.01), *** (*p* value ≤ 0.001). Right: confocal maximum intensity projection of cells on the 100 μm wide line (where the nuclei are shown in blue) illustrating the angular orientation of the different nuclei, where one negatively and one positively oriented nucleus is enlarged. To obtain the data, three hydrogel samples were utilized per line width, where four images were acquired and analysed per hydrogel sample, which equates to 12 images per line width in total. The number of cells analysed for each line width varied between 400 for the 10 μm wide lines and 1400 for the 400 μm wide lines. Scale bars = 100 μm. Created with https://BioRender.com.

Cellular chirality bias, and conversely cell orientation, has previously been measured in different ways *e.g.*, by studying the cell body orientation, nucleus orientation, or the orientation of other organelles in relation to each other.^15,16,18^ Here, we measured both the cell body (as depicted by ZO-1 staining in the cell membrane) and the nucleus (as depicted by Hoechst staining) orientation and compared that to the micropatterned line direction. From our previous study investigating the alignment of brain microvascular endothelial cells, we had not found any statistically significant differences between the alignment of the cell body and the nucleus, on a population level, and these two measurement are essentially interchangeable.^21^ However, we still wanted to make sure this was true when it came to the chirality bias as well. Hence, we investigated the chirality bias of both the cell body and the nucleus and found that there were no statistically significant differences between these two measurements on the same line width, even though it was noted that the cell body chirality measurements displayed a more pronounce trend as the lines’ width increased (Fig. 2A).

By looking at the percentage of cells exhibiting a negative or positive chirality bias on the different micropatterned line widths it could be seen that the cells exhibited their most pronounced bias at the 100 μm wide lines for both the cell body and the nucleus as compared to the cells seeded on the non-patterned hydrogels (*p* value ≤ 0.001) (Fig. 2A, ESI Tables 1 and 2†). It could also be observed that going from the 10 μm to 100 μm wide lines, the exhibited chirality bias increased more and more. At the 200 μm wide line the chirality bias seemed to be decreasing again, while still being exhibited. On the other hand, cells on the 300 μm and 400 μm wide lines did not seem to be exhibiting any chirality bias compared to cells seeded on the non-patterned hydrogels, and we did not find any statistically significant difference between theses either.

Finding the most pronounced chirality bias on the 100 μm wide lines is also supported when looking at the mean orientation of the cells. Not only are more cells negatively oriented on these lines but the mean orientation away from 0° is also highest here, with a mean orientation angle compared to the line direction of −9.19° (Fig. 2B, ESI Tables 3 and 4†). When the line width is decreased to 10 μm, the cells’ mean orientation angle is instead approaching 0° (0.14°). When the line width is instead approaching infinity from the cell’s perspective, on the non-patterned hydrogels, the mean orientation angle even becomes positive (1.73°). Statistically the mean orientation of the cells on the 100 μm wide lines are significantly different from the cell mean orientations on all but the 75 μm and 200 μm wide lines.

For the nuclei orientations, this trend is not as evident and even reverts, as the orientation away from 0° instead increases, going from the 25 μm line and approaching the 10 μm wide line (ESI Tables 5 and 6†). Even so, the nuclei orientation still confirms that brain microvascular endothelial cells on the 100 μm wide lines exhibit the most pronounce chirality bias, and the nucleus mean orientation on the 100 μm wide lines also showed a statistically significant difference compared the nucleus mean orientation on all other lines except the 75 μm and 200 μm wide lines, as seen for the cell mean orientations as well. In addition, there was no statistically significant difference between the cell and the nucleus mean orientation on these lines (*p* = 0.547).

While the cells on the 10 μm wide lines are strongly oriented with the line direction, they experience less freedom to move, both their cell body and their nucleus, and exhibit their chirality bias. This is likely due to these cells receiving simultaneous orientation cues from touching both appositional boundaries. Hence, these cells cannot exhibit their fully pronounce chirality profile.

The cells on the wider lines, as well as the non-patterned hydrogels, on the other hand, don’t receive enough orientation cues to exhibit any pronounced chirality bias. On these wider lines or areas, only the cells close to the line boundaries receive directional cues, but these don’t reach the cells in the line centre. The directional cues from the line boundary are propagated inwards to neighbouring cells. However, the directional information is usually only propagated a few cells inwards from the boundary, and the propagation length is highly cell type dependent.^14^ The 100 μm wide lines here provide just the right balance between directional cues and freedom to move for the brain endothelial microvascular cells to allow them to exhibit their most pronounced chirality bias. These results give important implications as a suboptimal pattern width, leading to suboptimal exhibition of chirality bias, might have detrimental effects on further experimental results. Small changes in cell chirality may go undetected if suboptimal micropattern sizes are used. This might be detrimental when studying the effects of *e.g.*, disease states or toxic substances on cell chirality, where such small changes, such as randomization or a reduced exhibited cell chirality bias might be the result.

### The cell chirality of brain microvascular endothelial cells is dependent on the cell’s location on the micropatterned line

**Fig. 3:**
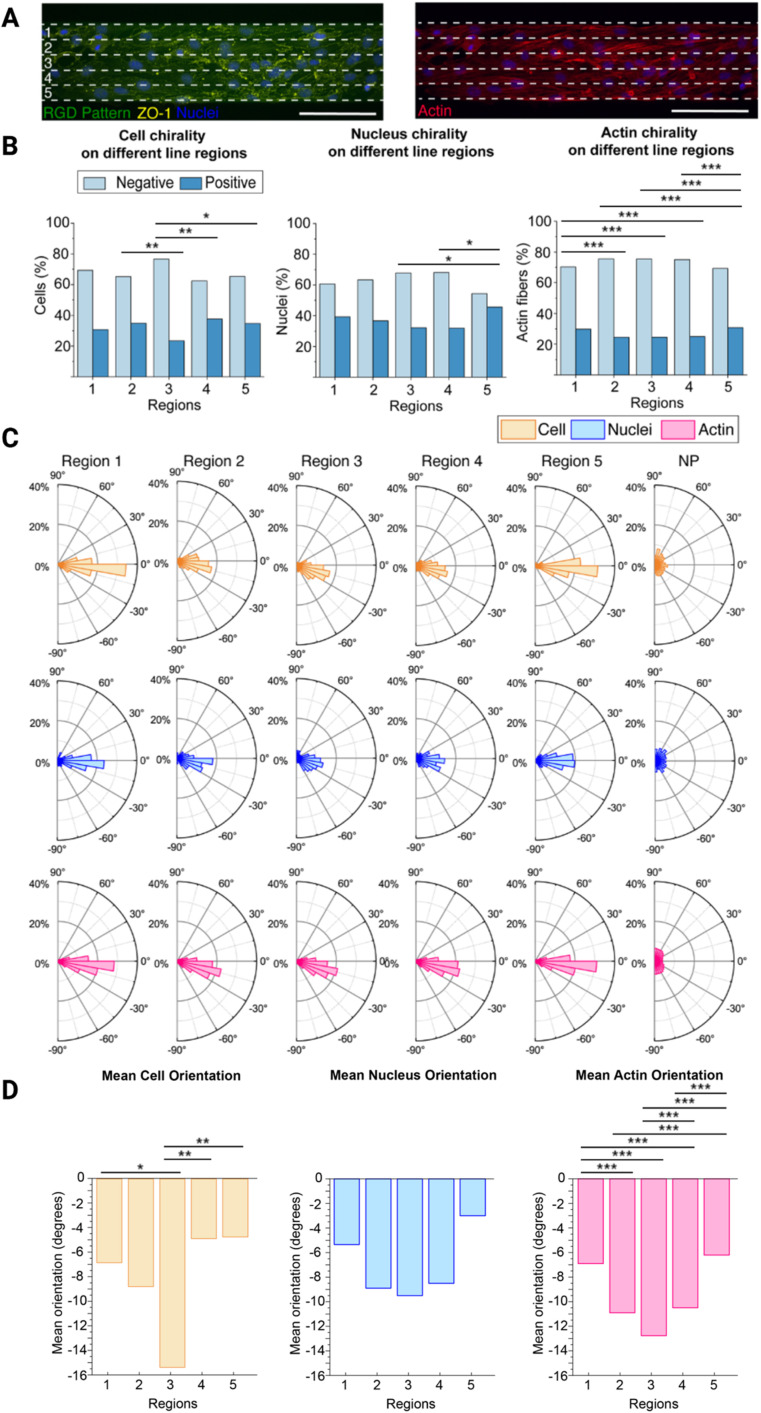
Brain microvascular endothelial cell chirality propagation from the boundary. (A) Representative image of cells, stained for ZO-1 (yellow) and nucleus (blue) (left), and F-actin (red) (right), on the 100 μm wide line patterns (green), divided into five equal regions (1–5). The white dashed lines indicate the five resulting regions, each 20 μm wide. These regions (1–5) in the schematic corresponds to the regions in B–D. (B) Bar plots with the percentage of cells, nuclei, and F-actin fibres exhibiting a chirality bias (negative chirality bias in light blue and positive chirality bias in dark blue) within each of the five regions (1–5) depicted in (A). Statistical analysis of cell, nucleus, and actin orientation of brain microvascular cells in the different regions were assessed using a chi-square test. Significance is symbolized by non-significant or N.S. (*p* > 0.05), * (*p* ≤ 0.05), ** (*p* value ≤ 0.01), *** (*p* value ≤ 0.001). (C) Polar plots indicating the orientation of the cells, nuclei, and F-actin fibres of cells in the five regions (1–5) as depicted in (A), and the non-patterned (NP) area, included as a comparison. (D) Bar plots showing the mean orientation of the cells, nuclei, and F-actin fibres of the different regions (1–5) depicted in (A). Statistical analysis of cell, nucleus, and actin chirality of brain microvascular endothelial cells in the different regions (1–5) were determined using a one way analysis of variance (ANOVA) with a Games-Howell *post hoc* for multiple comparisons at a 95% confidence level. Significance is symbolized by non-significant or N.S. (*p* > 0.05), * (*p* ≤ 0.05), ** (*p* value ≤ 0.01), *** (*p* value ≤ 0.001). To obtain the data, three hydrogel samples were utilized per line width, where four images were acquired and analysed per hydrogel sample, which equates to 12 images in total. The total number of cells analysed was around 900. Scale bars = 100 μm. Created with https://BioRender.com.

Next, we further wanted to understand how the cell chirality bias is exhibited across the line width. To do this, we used the 100 μm wide lines as the cells exhibited the most pronounced chirality bias on these lines. We divided the 100 μm wide lines into five equal regions (each 20 μm wide) and observed how the cells behaved in each region (Fig. 3A). There are generally 5–10 cells spanning the entire 100 μm width. Hence, in each 20 μm wide subregion of these lines, there should be 1–2 cells in parallel. Along with the cell and nucleus chirality previously investigated, we decided to also investigate F-actin chirality variability over the regions, as actin dependent mechanisms have been reported to initiate the establishing of chiral properties.^25^

Overall, both the cells, nuclei, and F-actin fibres are exhibiting a negative chirality bias in all five regions, however, this bias is more pronounced in region 3 located in the centre on the lines (Fig. 3B, ESI Tables 7–9†). On the opposite, the exhibited chirality is less pronounced in regions 1 and 5 located next to the line boundaries, where region 5 seems to have the lowest exhibited chirality bias. This is supported by the fact that both the cell and nucleus chirality bias was found to be statistically different from the centre region (3), while region 1 was not. The actin fibre chirality bias is also more pronounce in the centre regions (2–4) as these are statistically significantly different from the side regions (1 and 5). As there are more actin fibres than cells and nuclei smaller differences between these regions also becomes statistically significantly different as *n* increases. It can also be seen that both cells, nuclei, and F-actin fibres are exhibiting a negative orientation bias, where again this is more pronounced in the centre region 3 (Fig. 3C). In this centre region the mean orientation away from 0° of both the cells, nuclei, and F-actin fibres is also most pronounced and in line with the rest of the data (Fig. 3D, ESI Tables 10–15†). This is also supported by the presence of a statistically significant difference between the side regions (1 and 5) and the centre region (3) for the cell mean orientation, however, no statistically significant difference was visible on the mean nucleus orientation. We also observed a pronounced statistically significand differences in the actin fibre orientation between the different regions.

These differences can be explained by their distance from the outer boundary of region 1 and outer boundary of region 5. In region 3, in the centre of the line, the cells still receive directional cues from the cells closer to the boundaries, but they also have more freedom to move and exhibit their chirality bias. In region 1 the cells are restricted by the boundary on one side and free to move on the other. However, as they are preferably turning to the left, or towards the outer boundary of region 5, as they are negatively biased, they still have the freedom to move in this preferred direction and are thus only partly hindered to exhibit their preferred chirality bias. The cells in region 5, on the other hand, are restricted by the boundary on the other side. In this case, they are more hindered to exhibit their preferred chirality bias as they want to turn towards the region 5 outer boundary, but are hindered by the boundary.

Understanding the presence of differences along the line width is important when designing chirality experiments. Rather than analysing all cells, perhaps the cells along the outer boundaries should not be include in the analysis. Since these cells are more sterically hindered to exhibit their preferred chirality bias, by the presence of the boundary, subtill difference in experimental outcomes might be masked when including these cells in analysis, especially on the narrower lines where *de facto* a higher ratio of the cells will be closer to the boundaries. Choosing the optimal pattern width and including only the centre cells in the analysis may thus provide more physiologically relevant chirality experiment results.

### The ZO-1 expression of brain microvascular endothelial cells is dependent on the cell’s location on the micropatterned line

ZO-1 is a tight junction adaptor protein, that it is essential for barrier formation between brain microvascular endothelial cells, which anchors the tight junctions to the actin cytoskeleton, stabilizing and maintaining barrier integrity.^26–28^ It has previously been shown that the ZO-1 intensity decreases when there is a chirality mismatch between neighbouring cells that leads to poor junction formation.^13^ Since we observed exhibited chirality bias differences along the width of the 100 μm wide lines, we wanted to investigate if the ZO-1 expression varies along the line width as well.

**Fig. 4:**
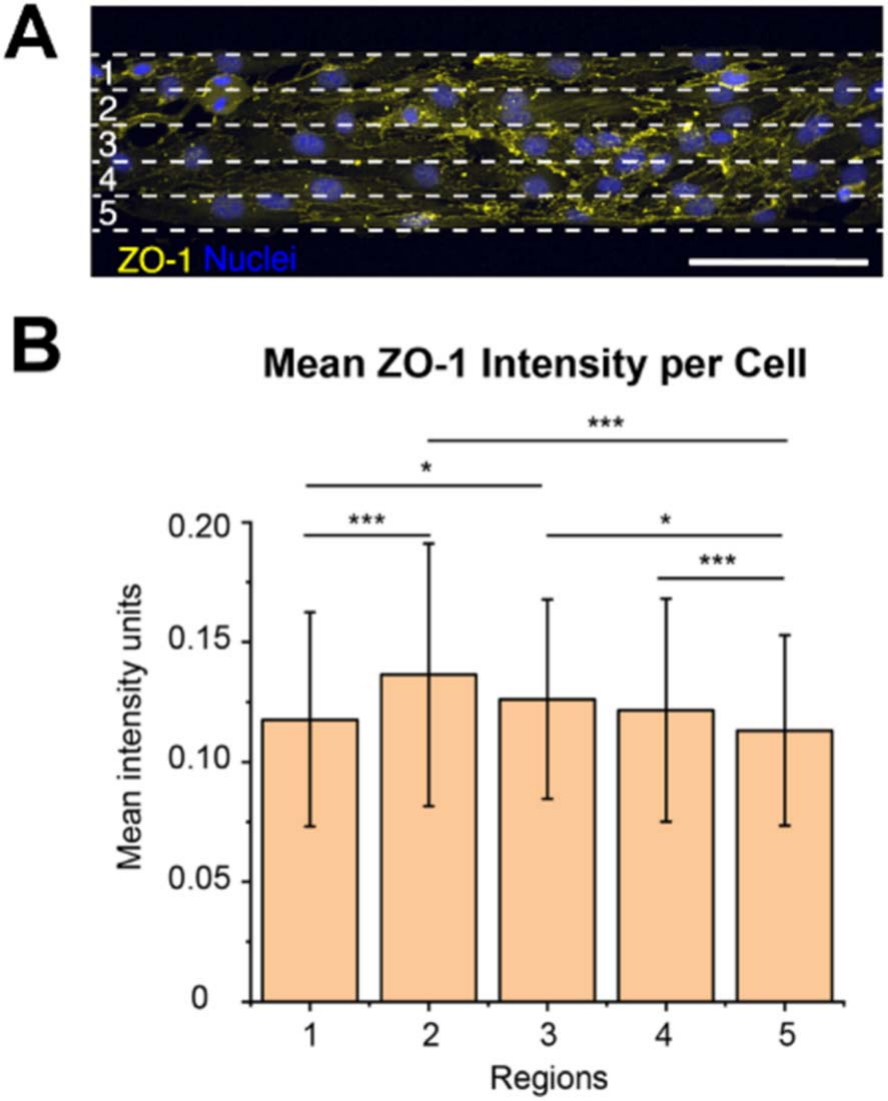
ZO-1 expression depending on brain microvascular endothelial cell location. (A) Representative image of cells, stained for ZO-1 (yellow) and nucleus (blue), on the 100 μm wide line patterns, divided into five equal regions (1–5). The white dashed lines indicate the five resulting regions, each 20 μm wide. These regions (1–5) in the schematic corresponds to the regions in (B). (B) Bar plot of the mean intensity units of ZO-1 per cell within each of the five regions (1–5) depicted in (A). The statistical significance of the ZO-1 mean intensity in the different regions was determined by performing a one way analysis of variance (ANOVA) with a Games-Howell *post hoc* for multiple comparisons at a 95% confidence level. The statistical significance is symbolized by * (*p* ≤ 0.05), ** (*p* value ≤ 0.01), *** (*p* value ≤ 0.001). To obtain the data, three hydrogel samples were utilized per line width, where four images were acquired and analysed per hydrogel sample, which equates to 12 images per line width in total. The total number of cells analysed was around 900. Scale bars = 100 μm.

We measured the mean intensity units of ZO-1 per cell in the different regions (1–5, Fig. 4A). It could be seen that the ZO-1 intensity per cell is higher in the three centre regions (2–4) than in the edge regions (Fig. 4B, ESI Table 16†). This is naturally so, because the cells in the centre regions have more cell–cell contact, while the cells on the boundaries don’t have any cells to form junctions with along the boundaries. As the cells along the boundaries are also more hindered to exhibit their chirality bias more mismatches might occur here with their neighbours on the centre side. Chirality mismatches, where neighbouring cells’ exhibited chirality is not matching, leads to poorer junction formation and thus less ZO-expression.

We also observed that the ZO-1 intensity per cell seems to be the highest in region 2, however, there was no statistically significant difference between the three centre regions (2–4). As region 2 is closer to the outer boundary of region 1 than to the outer boundary of region 5, they are less hindered to exhibit their preferred chirality bias and turn in the direction of the outer boundary of region 5, than the cells in regions closer to the outer boundary of region 1. This might result in less chirality mismatches and thus a higher ZO-1 expression in this region. However, this is not the region in where we observed the most pronounced chirality bias exhibition, as was seen in region 3. We hypothesize that even though the cells in region 3 exhibits a more pronounced chirality bias this region has more mismatches against the cells in region 4, leading to less ZO-1 expression in this region. As the cells in region 4 are situated closer to the outer boundary of region 5 they are more sterically hindered to turn towards this boundary and fully exhibit their preferred chirality bias. This might then create mismatches against the cells in region 3 which are freer to turn and exhibit their chirality bias.

## Conclusions

In this paper we demonstrated the effect of the micropatterned line width on the exhibition of cell chirality bias of brain microvascular endothelial cells. We observed that cell chirality bias was most pronounced on 100 μm wide lines, where the cell alignment had a negative bias. Furthermore, we observed that the exhibition of cell chirality, and connected cell–cell junction expression, is dependent on the cell’s location on the micropatterned line.

When conducting cell chirality studies is it important to optimize the pattern width to where the cells exhibit the most chirality bias. If too small patterns are used, the cells’ freedom to move is constrained, as the cells receive alignment information from both boundaries. This leads to that the cells cannot exhibit their fully pronounced chirality bias. When cells are seeded on too large patterns there is instead a decrease in exhibited chirality bias, as the transmission of the boundary alignment information cannot propagate between all cells across the whole pattern. At the optimal pattern width, the balance between the two boundaries is right, and the cells can exhibit their fully pronounced chirality bias, while still being aligned by the micropattern. In addition, based on the wide difference in chirality bias exhibition along the line width, cells along the boundaries, that are more sterically hindered to exhibit their preferred chirality bias might compromise a chirality-based analysis when small induced changes might be masked by their presence. Finding the optimal micropattern width and include only the cells in the centre of the micropattern in the analysis for chirality-based experiments might have a great impact on further experimental results.

This work serves as a guide to determine the optimal micropattern width and analysis area for further investigations where cell chirality bias might be lost due to substances causing cell dysfunction or disease.

## Materials and methods

Parts of the raw image data was produced for another purpose as previously reported^21^ including data available in.^29^ These images were here reused and analysed in a different way as well as complemented with the preparation and analysis of cells cultured on 200, 300 and 400 μm wide lines.

### Preparation and micropatterning of hyaluronic acid-acrylamide hydrogels

Sodium hyaluronate was modified with acrylamide groups (14% degree of functionalization) to form crosslinked hydrogels that could be micropatterned with RGD peptides, as previously reported.^30^ Briefly, the photoinitiator Irgacure 2959 (Sigma) was dissolved to a final concentration of 0.4% (w/v) in phosphate buffer. Freeze dried hyaluronic acid-acrylamide (HA-am) was dissolved in the photo-initiator solution to a final concentration of 2.0% (w/v). The HA-am solution was placed in a Si/SU8 mould to control the size and shape of the resulting hydrogel and covered by a methacrylate-functionalized glass coverslip. The system was then exposed to UV light (4.6 J cm^−2^) resulting in a crosslinked hydrogel, 8 mm in diameter and 200 μm thickness on the glass. Then, the hydrogel was covered by a 0.5 mM solution of 5-FAM-GCGYRGDSPG peptide (Innovagen AB). A photomask was then placed on top, and the system was exposed to UV light (1.9 J cm^−2^). The photomasks consisted of line openings of varying widths (10, 25, 50, 75, 100, 200, 300 and 400 μm) with 100 μm spacing between openings.

### Cell culture

Mouse brain microvascular endothelial cells (bEnd.3 ATCC) were cultured in Dubelcco’s modified eagle medium high glucose (Gibco) supplemented with 10% foetal bovine serum (Cytiva) and 1% penicillin streptomycin (Lonza) and maintained at 37 °C and 5% CO_2_. Cell media was renewed every two days and the cells were passaged when reaching 80% confluency using TrypLE express (Gibco).

Chirality studies consisted in seeding bEnd.3 cells (passage between 24 and 35) on the patterned hydrogels (lines 10, 25, 50, 75, 100, 200, 300 and 400 μm in width) at a seeding density of 20 000 cells per cm^2^. The cells were cultured on the at 37 °C in 5% CO_2_ for 24 h until they were fixed and analysed.

### Immunofluorescence staining of patterned cells

After 24 h in culture, the cells were fixed using 2% paraformaldehyde in PBS (Biotium). The cell samples were then blocked for 2 h with a blocking buffer (3% bovine serum albumin (BSA) and 0.2% IGEPAL (Sigma) in PBS). Zonula occludens-1 (ZO-1) proteins were stained using a rabbit ZO-1 primary antibody (Invitrogen) and goat anti-rabbit Alexa Fluor 568 (Invitrogen), followed by nuclei staining using Hoechst 33342 (Invitrogen). Where F-actin was stained, SPY620-actin (Spirochrome) was used.^21,29^

The ZO-1 primary antibody was dissolved (1 : 100) in 50% blocking buffer in PBS and incubated overnight at 4 °C. The samples were then washed three times with Triton X-100 (0.3%). The goat anti-rabbit Alexa Fluor 568 antibody was dissolved in 50% blocking buffer in PBS and incubated for 2 h at room temperature (RT). The F-actin staining was performed using SPY620-actin and incubating for 1 h at RT followed by nuclei counterstaining with Hoechst 33342 (2000X) and incubating for 20 min at RT.

### Image acquisition

Confocal stacks were acquired with a laser scanning confocal microscope (Leica SP8) equipped with a 25X/0.95 water immersion objective. The z-stacks were collected from the samples with a fixed distance of 0.57 μm between the slices, as deemed to be optimal by the microscopy software. The stacks were then projected into maximum intensity projections using Fiji (ImageJ).^31^

### Cell chirality image analysis

To obtain information of the cell chirality of the bEnd.3 cells on the line patterns, three hydrogel samples were utilized per line width, where four images were acquired and analysed per hydrogel sample, which equates to 12 images per line width in total. The number of cells analysed for each line width varied between 400 for the 10 μm wide lines and 1400 for the 400 μm wide lines.

Cell alignment to the line micropattern was calculated using CellProfiler™,^20^ as previously described.^21^ Briefly, the nuclei were segmented and identified using the IdentifyPrimaryObject module on the nuclei image. Next, the cell was segmented using the nuclei as a seed in IdentifySecondaryOject on the ZO-1 image. Then, the cell and nuclei orientation were calculated using the MeasureObjectAreaShape module, where the objects are fitted into an ellipse. Then, the angle between the major axis of the ellipse and the *x*-axis, determined by direction of the underlying RGD line pattern, was calculated. The orientation values range between ±90°. An object whose major axis is oriented between −90° and 0° was deemed as negative, and an object that orientated between 0° and 90° was deemed positive (Fig. 1). The negative and positive directions are determined by the numpy coordinate space, where the coordinate origin is situated at the top left of the image.

To determine the cell chirality regionality data was obtained from analysing 12 images, collecting four images each from three different hydrogels patterned with the 100 μm wide lines which were divided into five separate 20 μm wide regions. The total number of cells analysed was around 900.

### ZO-1 intensity measurements

The mean intensity units from the ZO-1 image per cell were determined using the MeasureObjectIntensity module in CellProfiler™, where the mean ZO-1 intensity is measured for each cell object, identified in the previous modules. These experiments on 100 μm wide lines resulted in 12 images used for this analysis. Cells were divided into five different regions as previously described.

### Statistical analysis

Statistical analysis was performed on Mintab 18. Statistical analysis of cell chirality of brain microvascular cells on different line widths compared to cells culture on the non-patterned surfaces was assessed using a chi-square test.

Statistical analysis of cell, nucleus, and actin orientation of brain microvascular cells in the different regions were assessed using a chi-square test.

One way analysis of variance (ANOVA) with a Games-Howell *post hoc* for multiple comparisons at a 95% confidence level was used to compare the mean ZO-1 expression per cell on the different line regions.

The significance is symbolized by non-significant or N.S. (*p* > 0.05), * (*p* ≤ 0.05), ** (*p* value ≤ 0.01), *** (*p* value ≤ 0.001).

## Author contributions

MA and MT conceived, planned and supervised the study, performed data analysis, wrote part of and edited the full manuscript, AMPH performed the experimental work, performed data analysis and wrote part of the manuscript. All authors read and approved the final version of the manuscript.

## Conflicts of interest

There are no conflicts to declare.

## Supplementary Material

RA-012-D2RA05434E-s001
