# Supplementary material for: Cell chirality exhibition of brain microvascular endothelial cells is dependent on micropattern width
Source: RSC Adv. 2022 Oct 21;12(46):30135–44. doi: 10.1039/d2ra05434e (PMC9585451; doi:10.1039/d2ra05434e)
Supplement: RA-012-D2RA05434E-s001 [file RA-012-D2RA05434E-s001.pdf]

Electronic Supplementary Material (ESI) for RSC Advances.
This journal is © The Royal Society of Chemistry 2022

Cell chirality exhibition of brain microvascular endothelial cells is dependent on micropattern width.

Ana María Porras Hernández[1], Maria Tenje[1,*], Maria Antfolk[2, 3,*]

[1]Dept. of Materials Science and Engineering, Science for Life Laboratory, Uppsala University, Uppsala, Sweden.
[2]Dept. of Biomedical Engineering, Lund University, Lund, Sweden.
[3]Biotech Research and Innovation Centre, University of Copenhagen, Copenhagen, Denmark

Corresponding authors: Maria Antfolk (maria.antfolk@bme.lth.se), and Maria Tenje (maria.tenje@angstrom.uu.se)

*M.T and M.A jointly supervised this work

Supplementary Information

Supplementary Table 1. Statistical analysis of cell chirality of brain microvascular endothelial cells on different line widths compared to cells seeded on non-patterned hydrogels. The statistics were assessed using a chi-square test. Significance is symbolized by non-significant or N.S. (p>0.05), * ($p \leq 0.05$), ** (p value ≤ 0.01), *** (p value ≤ 0.001).

Difference of Levels	Chi-Square	DF	P- value	
10 – NP	1.087	1	0.297	N.S.
25 – NP	1.691	1	0.193	N.S.
50 – NP	4.302	1	0.038	*
75 – NP	25.116	1	<0.0001	***
100 – NP	44.129	1	<0.0001	***
200 – NP	3.271	1	0.071	N.S.
300 – NP	0.214	1	0.644	N.S.
400 – NP	2.822	1	0.093	N.S.

Supplementary Table 2. Statistical analysis of nucleus chirality of brain microvascular endothelial cells on different line widths compared to cells seeded on non-patterned hydrogels. The statistics were assessed using a chi-square test. Significance is symbolized by non-significant or N.S. (p>0.05), * ($p \leq 0.05$), ** (p value ≤ 0.01), *** (p value ≤ 0.001).

Difference of Levels	Chi-Square	DF	P- value	
10 – NP	4.435	1	0.035	*
25 – NP	3.663	1	0.056	N.S.

50 – NP	0.275	1	0.6	N.S.
75 – NP	11.629	1	0.001	***
100 – NP	47.462	1	<0.0001	***
200 – NP	7.325	1	0.007	**
300 – NP	0.757	1	0.757	N.S.
400 – NP	0.392	1	0.531	N.S.

Supplementary Table 3. Cell orientation descriptive statistics associated with Fig 2B.

**Line width (µm)**	**Mean (°)**	**Standard Deviation (°)**	**Median (°)**
10	0.137	15.259	-0.264
25	-0.967	17.327	-0.97
50	-1.629	23.813	-1.593
75	-5.729	27.145	-5.031
100	-9.19	34.7	-8.32
200	-2.54	42.0	-3.68
300	0.05	43.74	0.82
400	-1.93	47.58	-5.43
NP	1.73	51.85	0.37

Supplementary Table 4. Statistical analysis of cell chirality of brain microvascular endothelial cells on different line widths and cells seeded on non-patterned hydrogels. One way analysis of variance (ANOVA) was used with a Games-Howell post hoc for multiple comparisons at a 95% confidence level. Significance is symbolized by non-significant or N.S. ($p>0.05$), * ($p \leq 0.05$), ** (p value ≤ 0.01), *** (p value ≤ 0.001). The data is presented as mean and standard deviation.

**Difference of Levels**	**Difference of Means**	**SE of Difference**	**95% CI**	**T-Value**	**Adjusted P-Value**	
10 – 25	1.104	1.076	(-2.241,4.449)	1.026	0.9834	N.S.
10 – 50	1.766	1.210	(-1.997,5.528)	1.459	0.8742	N.S.
10 – 75	5.866	1.242	(2.005,9.727)	4.721	<0.0001	***
10 – 100	8.611	1.208	(4.859,12.36)	7.131	<0.0001	***
10 – 200	2.677	1.967	(-3.443,8.798)	1.361	0.9119	N.S.
10 – 300	0.08735	1.956	(-5.997,6.172)	0.04466	>0.9999	N.S.
10 – 400	2.064	1.445	(-2.425,6.552)	1.428	0.8872	N.S.
10 – NP	-1.594	1.874	(-7.416,4.229)	0.8505	0.9952	N.S.
25 – 50	0.6616	1.221	(-3.132,4.455)	0.5421	0.9998	N.S.
25 – 75	4.762	1.252	(0.8705,8.653)	3.802	0.0047	**
25 – 100	7.507	1.218	(3.724,11.29)	6.165	<0.0001	***
25 – 200	1.573	1.973	(-4.566,7.713)	0.7973	0.9969	N.S.

25 – 300	-1.017	1.962	(-7.121,5.087)	0.5180	0.9999 N.S.
25 – 400	0.9598	1.454	(-3.555,5.474)	0.6602	0.9992 N.S.
25 – NP	-2.698	1.880	(-8.540,3.145)	1.435	0.8844 N.S.
50 – 75	4.100	1.370	(-0.1550, 8.355)	2.994	0.0693 N.S.
50 – 100	6.846	1.338	(2.689, 11.00)	5.116	<0.0001 ***
50 – 200	0.9118	2.050	(-5.464, 7.287)	0.4448	>0.9999 N.S.
50 – 300	-1.678	2.039	(-8.019, 4.663)	0.8230	0.9962 N.S.
50 – 400	0.2982	1.556	(-4.534, 5.130)	0.1916	>0.9999 N.S.
50 – NP	-3.359	1.960	(-9.450, 2.731)	1.714	0.7383 N.S.
75 – 100	2.746	1.367	(-1.501, 6.992)	2.008	0.5377 N.S.
75 – 200	-3.188	2.069	(-9.622, 3.246)	1.541	0.8358 N.S.
75 – 300	-5.778	2.058	(-12.18, 0.6217)	2.807	0.1146 N.S.
75 – 400	-3.802	1.581	(-8.711, 1.107)	2.405	0.2818 N.S.
75 – NP	-7.459	1.980	(-13.61, -1.307)	3.767	0.0054 **
100 – 200	-5.934	2.048	(-12.30, 0.4362)	2.897	0.0909 N.S.
100 – 300	-8.524	2.038	(-14.86, -2.188)	4.183	0.0010 **
100 – 400	-6.548	1.554	(-11.37, -1.723)	4.214	0.0009 ***
100 – NP	-10.20	1.959	(-16.29, -4.120)	5.210	<0.0001 ***
200 – 300	-2.590	2.562	(-10.55, 5.372)	1.011	0.9849 N.S.
200 – 400	-0.6137	2.197	(-7.442, 6.215)	0.2793	>0.9999 N.S.
200 – NP	-4.271	2.500	(-12.04, 3.495)	1.709	0.7413 N.S.
300 – 400	1.976	2.187	(-4.820, 8.773)	0.9037	0.9928 N.S.
300 – NP	-1.681	2.491	(-9.419, 6.057)	0.6748	0.9991 N.S.
400 – NP	-3.657	2.114	(-10.22, 2.906)	1.730	0.7277 N.S.

Supplementary Table 5. Nucleus orientation descriptive statistics associated with Fig 2B.

**Line width (µm)**	**Mean (°)**	**Standard Deviation (°)**	**Median (°)**
10	-1.91	19.78	-0.97
25	-0.97	17.33	-0.97
50	-0.49	35.26	0.09
75	-3.78	35.56	-4.02
100	-7.58	34.34	-8.23
200	-4.53	43.98	-8.09
300	-0.42	46.53	1.58
400	1.94	49.60	0.46
NP	2.04	51.82	2.48

Supplementary Table 6. Statistical analysis of nucleus chirality of brain microvascular endothelial cells on different line widths and cells seeded on non-patterned hydrogels. One way analysis of variance (ANOVA) was used with a Games-Howell post hoc for multiple comparisons at a 95% confidence level. Significance is symbolized by non-significant or N.S. ($p>0.05$), * ($p \leq 0.05$), ** (p value ≤ 0.01), *** (p value ≤ 0.001). The data is presented as mean and standard deviation.

**Difference of Levels**	**Difference of Means**	**SE of Difference**	**95% CI**	**T-Value**	**Adjusted P-Value**	
10 – 25	-0.9460	1.242	(-4.809, 2.918)	0.7615	0.9978	N.S.
10 – 50	-1.424	1.709	(-6.738, 3.890)	0.8331	0.9959	N.S.
10 – 75	1.864	1.621	(-3.175, 6.902)	1.149	0.9664	N.S.
10 – 100	5.669	1.503	(0.9991, 10.34)	3.772	0.0053	**
10 – 200	2.615	2.139	(-4.037, 9.267)	1.223	0.9514	N.S.
10 – 300	-1.492	2.154	(-8.192, 5.207)	0.6927	0.9989	N.S.
10 – 400	-3.855	1.614	(-8.869, 1.160)	2.388	0.2914	N.S.
10 – NP	-3.958	1.973	(-10.09, 2.172)	2.006	0.5392	N.S.
25 – 50	-0.4781	1.600	(-5.453, 4.497)	0.2988	>0.9999	N.S.
25 – 75	2.810	1.506	(-1.870, 7.489)	1.866	0.6376	N.S.
25 – 100	6.615	1.377	(2.336, 10.89)	4.802	<0.0001	***
25 – 200	3.561	2.052	(-2.825, 9.947)	1.735	0.7246	N.S.
25 – 300	-0.5464	2.069	(-6.982, 5.889)	0.2641	>0.9999	N.S.
25 – 400	-2.909	1.498	(-7.562, 1.744)	1.941	0.5850	N.S.
25 – NP	-3.012	1.879	(-8.852, 2.827)	1.603	0.8034	N.S.
50 – 75	3.288	1.910	(-2.645, 9.221)	1.722	0.7331	N.S.
50 – 100	7.093	1.810	(1.469, 12.72)	3.919	0.0030	**
50 – 200	4.039	2.365	(-3.311, 11.39)	1.708	0.7416	N.S.
50 – 300	-0.06825	2.379	(-7.462, 7.325)	0.02869	>0.9999	N.S.
50 – 400	-2.431	1.904	(-8.343, 3.482)	1.277	0.9381	N.S.
50 – NP	-2.534	2.216	(-9.418, 4.349)	1.144	0.9674	N.S.
75 – 100	3.805	1.727	(-1.559, 9.169)	2.203	0.4040	N.S.
75 – 200	0.7515	2.302	(-6.404, 7.907)	0.3265	>0.9999	N.S.
75 – 300	-3.356	2.316	(-10.56, 3.844)	1.449	0.8784	N.S.
75 – 400	-5.718	1.825	(-11.39, -0.05117)	3.133	0.0460	*
75 – NP	-5.822	2.149	(-12.50, 0.8520)	2.709	0.1451	N.S.
100 – 200	-3.053	2.220	(-9.955, 3.849)	1.375	0.9069	N.S.
100 – 300	-7.161	2.235	(-14.11, -0.2132)	3.204	0.0375	*
100 – 400	-9.523	1.721	(-14.86, -4.181)	5.535	<0.0001	***
100 – NP	-9.627	2.061	(-16.03, -3.226)	4.672	0.0001	***
200 – 300	-4.108	2.704	(-12.51, 4.296)	1.519	0.8466	N.S.

200 – 400	-6.470	2.297	(-13.61, 0.6696)	2.817	0.1116 N.S.
200 – NP	-6.574	2.562	(-14.53, 1.386)	2.566	0.2021 N.S.
300 – 400	-2.362	2.312	(-9.546, 4.821)	1.022	0.9838 N.S.
300 – NP	-2.466	2.575	(-10.47, 5.533)	0.9578	0.9894 N.S.
400 – NP	-0.1038	2.144	(-6.761, 6.553)	0.04841	>0.9999 N.S.

Supplementary Table 7. Comparative statistical analysis of cell chirality of brain microvascular endothelial cells in different regions (1-5 as seen in fig 3A) of the 100 µm wide line. The statistics were assessed using a chi-square test. Significance is symbolized by non-significant or N.S. ($p>0.05$), * ($p \leq 0.05$), ** (p value ≤ 0.01), *** (p value ≤ 0.001).

Difference of Levels	Chi-Square	DF	P- value
1 – 2	0.6579	1	0.417297 N.S.
1 – 3	2.2826	1	0.130832 N.S.
1 – 4	1.7637	1	0.184167 N.S.
1 – 5	0.4685	1	0.493662 N.S.
2 – 3	6.698	1	0.009652 **
2 – 4	0.3597	1	0.548685 N.S.
2 – 5	0.0014	1	0.970523 N.S.
3 – 4	9.8553	1	0.001693 **
3 – 5	4.9378	1	0.02627 *
4 – 5	0.3019	1	0.582706 N.S.

Supplementary Table 8. Comparative statistical analysis of nucleus chirality of brain microvascular endothelial cells in different regions (1-5 as seen in fig 3A) of the 100 µm wide line. The statistics were assessed using a chi-square test. Significance is symbolized by non-significant or N.S. ($p>0.05$), * ($p \leq 0.05$), ** (p value ≤ 0.01), *** (p value ≤ 0.001).

Difference of Levels	Chi-Square	DF	P- value
1 – 2	0.2533	1	.614783 N.S.
1 – 3	1.8367	1	.175341 N.S.
1 – 4	2.0153	1	.155718 N.S.
1 – 5	1.1117	1	.291721 N.S.
2 – 3	0.9346	1	.333677 N.S.
2 – 4	1.0761	1	.299575 N.S.
2 – 5	2.7341	1	.09823 N.S.
3 – 4	0.004	1	.94947 N.S.
3 – 5	6.0844.	1	.013638 *
4 – 5	6.4258	1	.011247 *

Supplementary Table 9. Comparative statistical analysis of actin chirality of brain microvascular endothelial cells in different line regions (1-5 as seen in fig 3A) of the 100 µm wide line. The statistics were assessed using a chi-square test. Significance is symbolized by non-significant or N.S. (p>0.05), * (p ≤ 0.05), ** (p value ≤ 0.01), *** (p value ≤ 0.001).

Difference of Levels	Chi-Square	DF	P- value
1 – 2	32.5211	1	< .00001 ***
1 – 3	32.0623	1	< .00001 ***
1 – 4	26.8299	1	< .00001 ***
1 – 5	0.6292	1	.427662 N.S.
2 – 3	0.0023	1	.961964 N.S.
2 – 4	0.3194	1	.571974 N.S.
2 – 5	40.3727	1	< .00001 ***
3 – 4	0.2682	1	.60455 N.S.
3 – 5	39.8817	1	< .00001 ***
4 – 5	34.2008	1	< .00001 ***

Supplementary Table 10. Cell orientation descriptive statistics associated with Fig 3D.

Regions	Mean (°)	Standard Deviation (°)	Median (°)
1	-6.79	17.57	-4.98
2	-8.76	30.46	-9.85
3	-15.34	31.08	-18.08
4	-4.87	32.28	-8.25
5	-4.73	19.93	-5.23

Supplementary Table 11. Statistical analysis of cell chirality of brain microvascular endothelial cells on different regions (1-5 as seen in Fig 3A). One way analysis of variance (ANOVA) was used with a Games-Howell post hoc for multiple comparisons at a 95% confidence level. Significance is symbolized by non-significant or N.S. (p>0.05), * (p ≤ 0.05), ** (p value ≤ 0.01), *** (p value ≤ 0.001). The data is presented as mean and standard deviation.

Difference of Levels	Difference of Means	SE of Difference	95% CI	T-Value	Adjusted P-Value
1 – 2	1,967	2,531	-4,971 to 8,906	0,7773	0,9370 N.S.
1 – 3	8,550	2,630	1,336 to 15,76	3,251	0,0110 *
1 – 4	-1,922	2,677	-9,265 to 5,421	0,7177	0,9524 N.S.
1 – 5	-2,059	2,309	-8,403 to 4,286	0,8915	0,8999 N.S.

2 – 3	6,583	2,985	-1,594 to 14,76	2,205	0,1796 N.S.
2 – 4	-3,889	3,027	-12,18 to 4,403	1,285	0,7007 N.S.
2 – 5	-4,026	2,706	-11,45 to 3,396	1,488	0,5713 N.S.
3 – 4	-10,47	3,110	-18,99 to -1,950	3,367	0,0074 **
3 – 5	-10,61	2,800	-18,29 to -2,930	3,789	0,0017 **
4 – 5	-0,1371	2,844	-7,937 to 7,663	0,04819	>0,9999 N.S.

Supplementary Table 12. Nucelus orientation descriptive statistics associated with Fig 3D.

Regions	Mean (°)	Standard Deviation (°)	Median (°)
1	-5.41	32.44	-4.82
2	-8.91	35.52	-9.43
3	-9.52	37.61	-14.31
4	-8.52	34.20	-10.30
5	-2.99	28.51	-2.26

Supplementary Table 13. Statistical analysis of nucleus chirality of brain microvascular endothelial cells on different regions (1-5 as seen in Fig 3A). One way analysis of variance (ANOVA) was used with a Games-Howell post hoc for multiple comparisons at a 95% confidence level. Significance is symbolized by non-significant or N.S. (p>0.05), * (p ≤ 0.05), ** (p value ≤ 0.01), *** (p value ≤ 0.001). The data is presented as mean and standard deviation.

Difference of Levels	Difference of Means	SE of Difference	95% CI	T-Value	Adjusted P-Value
1 – 2	3,498	3,636	-6,478 to 13,48	0,9621	0,8719 N.S.
1 – 3	4,106	3,797	-6,309 to 14,52	1,081	0,8161 N.S.
1 – 4	3,103	3,617	-6,823 to 13,03	0,8577	0,9120 N.S.
1 – 5	-2,429	3,730	-12,67 to 7,817	0,6511	0,9663 N.S.
2 – 3	0,6072	3,551	-9,121 to 10,34	0,1710	0,9998 N.S.
2 – 4	-0,3957	3,358	-9,595 to 8,804	0,1178	>0,9999 N.S.
2 – 5	-5,927	3,480	-15,47 to 3,621	1,703	0,4334 N.S.
3 – 4	-1,003	3,531	-10,68 to 8,672	0,2840	0,9986 N.S.
3 – 5	-6,534	3,647	-16,54 to 3,470	1,792	0,3801 N.S.
4 – 5	-5,531	3,460	-15,03 to 3,963	1,599	0,4993 N.S.

Supplementary Table 14. Actin orientation descriptive statistics associated with Fig 3D.

Regions	Mean (°)	Standard Deviation (°)	Median (°)
1	-6.95	22.70	-7.18
2	-11.45	25.74	-13.50
3	-12.71	26.76	-15.05
4	-10.52	24.93	-11.59
5	-6.22	21.12	-6.36

Supplementary Table 15. Statistical analysis of actin chirality of brain microvascular endothelial cells on different regions (1-5 as seen in Fig 3A). One way analysis of variance (ANOVA) was used with a Games-Howell post hoc for multiple comparisons at a 95% confidence level. Significance is symbolized by non-significant or N.S. (p>0.05), * (p ≤ 0.05), ** (p value ≤ 0.01), *** (p value ≤ 0.001). The data is presented as mean and standard deviation.

Difference of Levels	Difference of Means	SE of Difference	95% CI	T-Value	Adjusted P-Value
1 – 2	4,504	0,5057	3,125 to 5,884	8,906	<0,0001 ***
1 – 3	5,765	0,5151	4,360 to 7,171	11,19	<0,0001 ***
1 – 4	3,573	0,4977	2,215 to 4,931	7,180	<0,0001 ***
1 – 5	-0,7229	0,5094	-2,113 to 0,6670	1,419	0,6153 N.S.
2 – 3	1,261	0,5094	-0,1289 to 2,651	2,475	0,0963 N.S.
2 – 4	-0,9310	0,4918	-2,273 to 0,4107	1,893	0,3210 N.S.
2 – 5	-5,227	0,5036	-6,601 to -3,853	10,38	<0,0001 ***
3 – 4	-2,192	0,5014	-3,560 to -0,8239	4,371	0,0001 ***
3 – 5	-6,488	0,5130	-7,888 to -5,088	12,65	<0,0001 ***
4 – 5	-4,296	0,4955	-5,648 to -2,944	8,670	<0,0001 ***

Supplementary Table 16. Statistical analysis of ZO-1 intensity on different line regions (1-5 as seen in fig 4A) of the 100 µm wide line. One way analysis of variance (ANOVA) was used with a Games-Howell post hoc for multiple comparisons at a 95% confidence level. Significance is symbolized by non-significant or N.S. (p>0.05), * (p ≤ 0.05), ** (p value ≤ 0.01), *** (p value ≤ 0.001). The data is presented as mean and standard deviation.

**Difference of Levels**	**Difference of Means**	**SE of Difference**	**95% CI**	**T-Value**	**Adjusted P-Value**	
2 - 1	0.01868	0.00527	(0.00429, 0.03307)	3.54	0.004	***
3 - 1	0.00851	0.00476	(-0.00449, 0.02151)	1.79	0.381	N.S.
4 - 1	0.00393	0.00495	(-0.00959, 0.01744)	0.79	0.933	N.S.
5 - 1	-0.00455	0.00516	(-0.01864, 0.00954)	-0.88	0.904	N.S.
3 - 2	-0.01017	0.00469	(-0.02298, 0.00264)	-2.17	0.192	N.S.
4 - 2	-0.01475	0.00489	(-0.02809, -0.00142)	-3.02	0.021	*
5 - 2	-0.02323	0.00510	(-0.03714, -0.00931)	-4.56	<0.0001	***
4 - 3	-0.00458	0.00433	(-0.01640, 0.00723)	-1.06	0.828	N.S.
5 - 3	-0.01306	0.00457	(-0.02553, -0.00059)	-2.86	0.035	*
5 - 4	-0.00847	0.00477	(-0.02148, 0.00453)	-1.78	0.386	N.S.
